# GIS/GPS METHODOLOGIES TO MEASURING OUTDOOR MOBILITY, ENVIRONMENTAL CONTEXT, AND HEALTHY AGING

**DOI:** 10.1093/geroni/igad104.0905

**Published:** 2023-12-21

**Authors:** Christina Roecke, Minxia Luo, Jeffrey Kaye

## Abstract

There has been increasingly more interest in using GIS/GPS methodologies in healthy aging research, but insufficient consideration of the latest developments in this area. This symposium showcases recent studies using GIS/GPS methodologies to measure outdoor mobility and environmental context across different countries, including one long-term longitudinal study and three micro-longitudinal studies. Specifically, Lum and colleagues examine a four-year survey from over 2,000 older adults in Hong Kong. They show that public open space (including its accessibility, its number of leisure facility types, and social interactions happened therein) was not only associated with functional ability cross-sectionally, but also with the decline of functional ability over time. Perchoux and colleagues collect seven days data from 471 older adults in Luxembourg. They show that built environments (such as higher street connectivity and lower density of parking lots) were associated with lower total sedentary time. Luo and colleagues examine data from a 15-day study with 109 older adults in Switzerland. They show that characteristics of neighborhood walkability (including higher street connectivity, more access to places for walking and cycling, and lower mixture of land use) strengthens the positive associations between daily out-of-home mobility and daily working memory performance. Giannouli and colleagues present their project of a mobile app for early detection of mobility impairments. Through two one-week studies, they demonstrate the reliability, validity, and usability of their app as a tool to assess walking speed and life-space mobility of older adults. Finally, Jeffrey A. Kaye will discuss all papers from a healthy aging perspective.

